# A preliminary study on the online processing of anticipatory tonal coarticulation – Evidence from eye movements

**DOI:** 10.3389/fpsyg.2023.1137095

**Published:** 2023-04-20

**Authors:** Qian Li

**Affiliations:** Laboratory of Phonetics and Speech Science, Institute of Linguistics, Chinese Academy of Social Sciences, Beijing, China

**Keywords:** anticipatory tonal coarticulation, tone perception, eye-tracking, visual world paradigm, Tianjin Mandarin

## Abstract

While the f0 realization of lexical tones vary extensively in contexts, little has been known on how listeners process the variation in lexical tones due to contextual effects such as tonal coarticulation in spoken word recognition. This study thus aims to fill the knowledge gap in tone perception with evidence from two types of anticipatory tonal coarticulation effects in Tianjin Mandarin, i.e., the slope raising effect due to a following low-falling tone and the overall-height raising effect due to a following low-dipping tone. An eye-tracking experiment with the Visual World Paradigm was carried out to compare participants’ eye movements when they heard targets in three types of anticipatory raising conditions, i.e., the Slope Raising condition, the Overall-height Raising condition, as well as the No Raising condition (the baseline). The eye movement results showed significant differences in the proportion of looks to target between the Slope Raising condition versus the other two conditions, whereas the Overall-height Raising condition did not differ significantly from the No Raising condition. The findings thus suggest the facilitatory effect of tonal coarticulation cues in the anticipation of the upcoming tones, but listeners in this study seemed to be only sensitive to the raising in the f0 slope rather than the overall raising in the f0 height.

## 1. Introduction

In connected speech, the f0 realization of lexical tones may deviate largely from that produced in isolation, due to contextual effects such as the so-called phonological tonal sandhi and phonetic tonal coarticulation (see a review in Chen, 2012). However, while native speakers are known to utilize various f0 cues to identify lexical tones (e.g., Lin and Repp, 1989; Fox and Qi, 1990; Shen and Lin, 1991; Shen et al., 1993; Moore and Jongman, 1997), those studies on tone perception have not taken into consideration possible contextual f0 variation in connected speech, the perception of which is the most practiced behavior in speech communication.

So far, the very limited number of studies regarding tone perception in connected speech have mainly focused on the question of how listeners perceive lexical tones undergoing tone sandhi processes, or in other words, whether the sandhi-derived tones are identified or represented as another lexical tone within the tonal inventory of a language such as Standard Chinese (e.g., Wang and Li, 1967; Speer et al., 1989; Zhou and Marslen-Wilson, 1997; Chien et al., 2016; Meng et al., 2021; Yan et al., 2021; Tu and Chien, 2022). Far less attention has been paid to the perception of lexical tones that show f0 variation due to tonal coarticulation, which is however a more oft-seen phenomenon when tones are combined.

Moreover, in studies showing that the identification of coarticulated tones is dependent on the surrounding tonal contexts (e.g., Han and Kim, 1974; Lin and Wang, 1984; Xu, 1994), listeners were typically required to make offline judgments within the traditional meta-linguistic tonal identification paradigms. Only the end-state responses were recorded, which have been known to exhibit quite different patterns from the real-time processing in spoken word recognition (e.g., Spivey, 2008). While accumulated evidence has shown that just like segments, monosyllabic lexical tones are incrementally processed as the heard speech signals unfold (e.g., Malins and Joanisse, 2010; Yang and Chen, 2022; Zou et al., 2022), it is still unknown how listeners process the coarticulated tones, which are typically surfaced with distorted f0 and thus not the best exemplars of the tones as those over monosyllabic words.

Notably, in an eye-tracking study on anticipatory vowel nasalization in English using the Visual World Paradigm (VWP; Tanenhaus et al., 1995 and see a review in Huettig et al., 2011), Beddor et al. (2013) presented images of CVNC words (e.g., send) and CVC words (e.g., said) when listeners heard a [CṼNC] auditory target. The results showed that the listeners started to look at the target CVNC images even before the nasal consonants were heard. The listeners also directed earlier and more looks to the target CVNC image when the anticipatory nasalization occurred early in the vowel compared to that when the coarticulatory information occurred later. This has thus indicated that listeners are attentive to the fine-grained anticipatory coarticulatory information, and actively taking up the unfolding coarticulatory information as facilitatory cues in anticipating the upcoming nasal consonants. It is therefore reasonable to ask whether similar facilitatory effect of f0 variation due to contextual tonal coarticulation – especially the anticipatory tonal coarticulation – could also be observed in the supra-segmental domain. The present study thus aims to fill the knowledge gap on tone perception via a similar word-recognition task within the VWP. The testing case is Tianjin Mandarin, which is well-known for its complex tonal variation patterns over disyllabic and trisyllabic constituents.

Tianjin Mandarin is a dialect of Northern Mandarin, which is spoken in the urban areas of the Municipality of Tianjin, China (about 100 km from Beijing). Tianjin Mandarin has four canonical tones, which are cognates of those in Standard Chinese but are surfaced with different f0 realizations: Tone 1 (T1) in Tianjin Mandarin is a low-falling tone, Tone 2 (T2) a high-rising tone, Tone 3 (T3) a low-dipping tone, and Tone 4 (T4) a high-falling tone (Li et al., 2019). While previous impressionistic studies have claimed multiple tone sandhi patterns in disyllabic sequences for Tianjin Mandarin (e.g., Li and Liu, 1985; Chen, 2000; Hyman, 2007), three of them have been confirmed with experimental data, namely T1T1, T4T1, and T3T3 [Li and Chen, 2016; Li et al., 2019; but see (Wee et al., 2005; Zhang and Liu, 2011) for more tone sandhi patterns]. In all the three tonal combinations, the first tones show altered f0 realizations due to the following low tones. Specifically, the f0 directions of both falling tones T1 and T4 are inverted due to the following low-falling T1, and the low-dipping T3 is realized with a high-rising f0 contour due to the following T3.

In addition to the phonological sandhi alternations triggered by the following T1 and T3, these two low tones are also found to exert anticipatory tonal coarticulatory effects over the preceding lexical tones in two different ways (Li and Chen, 2016). Specifically, T1 induces greater f0 rise on the preceding tones (i.e., in T2T1 and T3T1), while T3 slightly raises the overall f0 height of the preceding tones (i.e., in T2T3 and T4T3).

The two different anticipatory effects thus present as good testing cases in examining how exactly fine-grained f0 variation affects the time course of processing and activation of lexical tones in online spoken word recognition. Two questions are of special interest here: (1) Does f0 variation over the first syllable due to anticipatory tonal coarticulation provide any facilitation in anticipating the second tone as similarly observed in segmental studies? (2) Are native listeners sensitive to f0 details such as different types of anticipatory raising in the real-time spoken word recognition?

Due to the fact that T1, T3, and T4 all involve tonal sandhi processes when followed by T1 or T3 in Tianjin Mandarin (i.e., T1T1, T3T3, and T4T1), a direct comparison between the two different coarticulatory raising effects can thus be made most clearly in the tonal context of T2Tx, where T2 is followed by different lexical tones. Therefore, three disyllabic tonal patterns were included for the target stimuli in the current study: (1) T2T1, where the first T2 shows a greater magnitude of f0 rising due to the following T1 (i.e., Slope Raising, hereafter as SR); (2) T2T3, where the first T2 is raised in the overall height by the following T3 (i.e., Overall-height Raising, hereafter as OR); (3) T2T4, where no raising effect is expected for the first T2 (i.e., No Raising, hereafter as NR). Listeners’ eye movements were recorded when they listened to the three types of target words and saw the corresponding printed words as opposing to a T2T2 competitor at the same time.

If anticipatory tonal coarticulation within the first tone indeed contributes to the anticipation of the second tone, it would be expected that the listeners direct earlier and/or more looks to the target words when they hear both the SR and the OR targets than when hearing a NR target. Furthermore, the two types of anticipatory raising effects would also give rise to different curves of proportions of looks if listeners are truly sensitive to the subtle differences in the manner of anticipatory raising.

## 2. Method

### 2.1. Participants

Thirty-three native speakers of Tianjin Mandarin (23 females; Mean = 21.9 years; SD = 1.7 years) participated in this experiment. All subjects were born and raised in the urban areas of Tianjin. They were undergraduate or postgraduate students studying in Beijing, but never lived out of Tianjin before the age of 18. They were paid for participation but unaware of the experiment purposes. All subjects had normal or corrected-to-normal vision. None of them reported any speech, hearing, or reading disorders.

### 2.2. Stimuli

The critical target stimuli consisted of 15 disyllabic words or lexicalized phrases where the first syllable was consistently T2, including three anticipatory tonal coarticulation conditions in Tianjin Mandarin: SR (i.e., T2T1), OR (i.e., T2T3), NR (i.e., T2T4), each with five items (as shown in the Supplementary material). These critical target stimuli were matched in bigram mutual information (Mean = 5.27, SD = 2.42, according to Da, 2004; corresponding to strong collocation strength). Results from ANOVA showed no significant difference among the three groups in bigram mutual information [*F*(2, 11) = 0.344, *p* = 0.717].

For each critical target, a corresponding T2T2 competitor (with segmental overlap with the target) and two phonologically unrelated distractors were chosen. The targets and competitors were further matched in lexical frequency (according to Cai and Brysbaert, 2010) and orthographic complexity (i.e., the number of strokes of the Chinese characters). Results of paired *t*-tests showed no significant difference between target and competitor for both frequency [*t*(14) = 0.27, *p* = 0.79] and orthographic complexity [*t*(14) = 0.96, *p* = 0.35].

**Figure 1:**
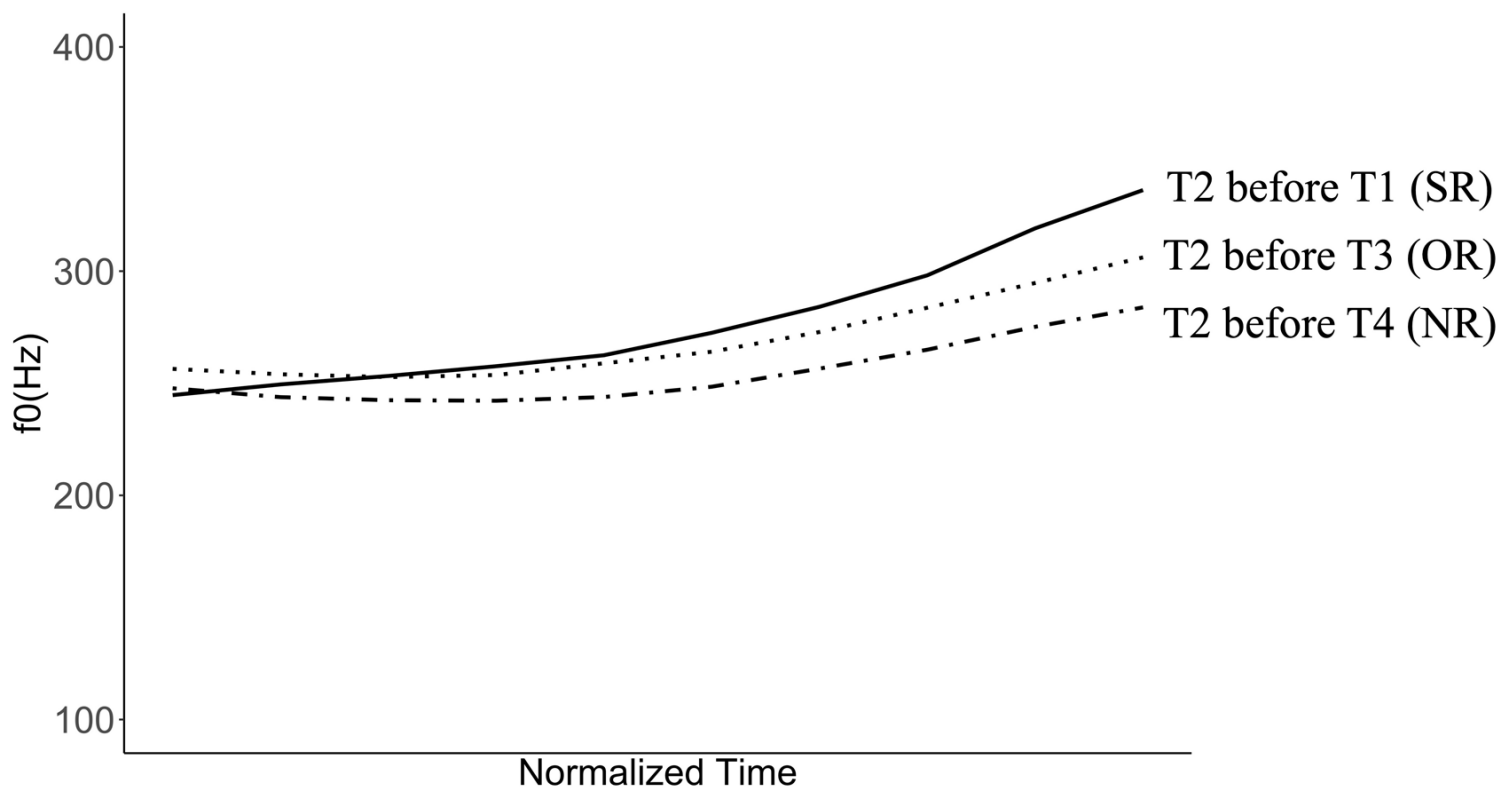
Mean f0 realization of T2 in three tonal coarticulation conditions averaged across five items in each condition. Solid line indicates the mean f0 of T2 before T1 (i.e., the SR condition), dotted line for T2 before T3 (i.e., the OR condition), dash-dot line for T2 before T4 (i.e., the NR condition). Normalized time.

All target stimuli were pre-recorded by a female speaker of Tianjin Mandarin who was born in the 1980s. The speaker was required to produce the target stimuli with the same loudness and speaking rate. All the stimuli were then manually segmented in Praat (Boersma and Weenink, 2020) for the extraction of syllable duration and f0 contours. The mean duration of the first syllable was 297 ms (SD = 82 ms) and was not significantly different across the three conditions [ANOVA: *F*(2, 12) = 1.43, *p* = 0.28]; the mean duration of the disyllabic stimuli was 761 ms (SD = 98 ms). Figure 1 plots the mean f0 realization of T2 over the first syllable of the disyllabic stimuli averaged across the five tokens within each condition. All f0 contours were normalized by taking 10 points (in Hz) with equal time interval in the rhyme part of the first syllable. As comparable to the prior findings in Li and Chen (2016), Figure 1 shows consistent rising f0 patterns of T2 regardless of the following lexical tones, while the T2 before T1 (i.e., the SR condition) shows the largest magnitude of f0 rising and the f0 of T2 before T3 (i.e., the OR condition) exhibits an overall height raising as compared with the T2 before T4 (i.e., the NR condition).

### 2.3. Procedure

Eye movement data in this study were recorded with an Eyelink 1000 system (SR Research, Ottawa, ON, Canada) with a 35 mm lens running at a 500 Hz sampling rate. Visual stimuli were presented on a 21-inch computer monitor (resolution: 1024 × 768 pixels). Participants were seated comfortably with a chin rest at 65 cm from the screen and were tested individually.

To get the participants familiar with the procedure, a practice block consisting of seven trials was implemented at the beginning. In addition to the 15 critical trials, 45 filler trials were also included. Each participant completed 60 trials in total. The trial order was conditionally randomized for each participant so that auditory stimuli of the same condition were not heard in consecutive trials.

**Figure 2:**
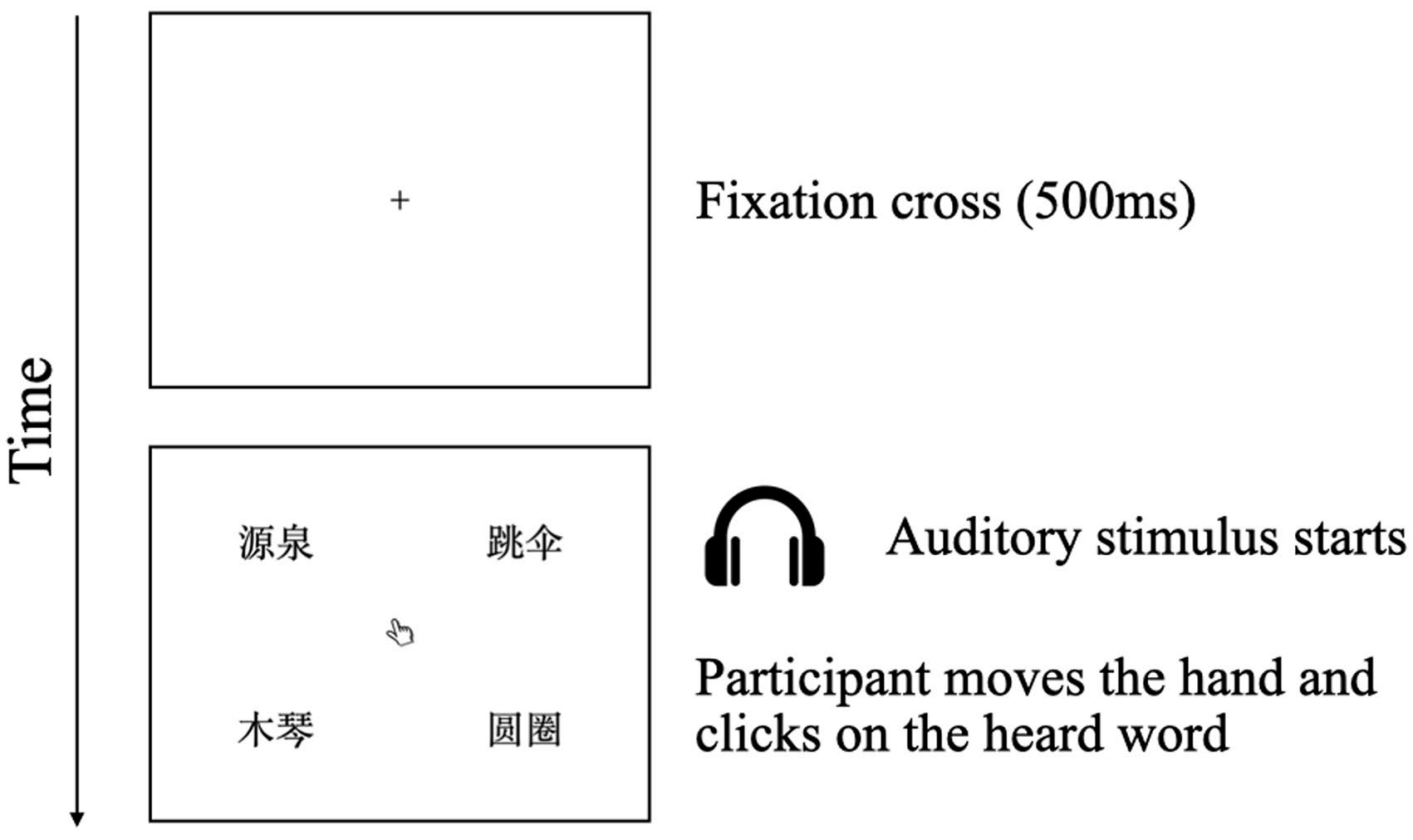
The procedure of the auditory and visual stimuli presentation.

In each trial, participants were shown with a fixation cross at the screen center for 500 ms. They were asked to look at the fixation cross until it disappeared and were then played with the auditory stimulus through a headphone simultaneously with the presentation of four printed words (i.e., Chinese characters) on the screen, i.e., a target (corresponding to the auditory stimulus), a competitor, and two distractors, as well as a mouse indicator (i.e., a hand image) at the very center of the screen. The task was to move the hand with the mouse and click on the heard word as soon as possible. An illustration of the stimulus presentation procedure is shown in Figure 2.

To guarantee the non-overlapping of the parafoveal view in the visual stimuli reading, each disyllabic word was located at the center of each of the screen quadrants (i.e., upper-left, upper-right, bottom-left, bottom-right) and fit into a 2.5 cm × 5 cm interest area so that the whole word maximally occupied a 5° visual angle (e.g., Miellet et al., 2009). Only fixations within interest areas were included as looks to the corresponding stimulus items.

The locations of the target visual stimuli on the screen were counterbalanced across trials, so that the target word appeared equal times in each location. Further fixation analyses also showed no significant difference across conditions in either the type of the first fixated Interest Area [Kruskal-Wallis test: χ^2^(2) = 5.84, *p* = 0.06] or the latency to that particular fixation since target onset [ANOVA: *F*(2, 491) = 0.04, *p* = 0.96].

### 2.4. Data analysis

The raw eye movement data (recorded at 2 ms intervals) were aggregated into 20 ms bins. The proportions of looks to different visual objects (i.e., target, competitor, and distractors) were calculated within each time bin and plotted from the stimulus onset to 1,600 ms post stimulus onset where the proportion of looks to target had reached the maximum (following Malins and Joanisse, 2010).

The response time (RT) for each trial was measured from the onset of auditory stimulus until the participant clicked on one of the four visual stimuli. The proportion data were fitted with orthogonal polynomials using Growth Curve Analysis (GCA) for statistics, in which the coefficients independently reflected the crucial characteristics of the curves (see Mirman, 2014 for details of GCA). Statistic modelling for both RT and the proportion data were carried out with the package lme4 (Bates et al., 2015) in R (R Core Team, 2022). Parameter-specific *p*-values were calculated via Satterthwaite’s degrees of freedom method using the lmerTest package in R (Kuznetsova et al., 2017).

## 3. Results

### 3.1. Behavioral results

The overall rate of correct responses for all trials reached the ceiling (i.e., 99.95%). Only one trial in the NR condition was made with an incorrect response, which was thus excluded from further analyses of eye movement data. Results of the linear mixed-effects modelling showed no significant general effect of TONAL CONDITION on RT [χ^2^(2) = 4.04, *p* = 0.13].

### 3.2. Eye movement results

**Figure 3:**
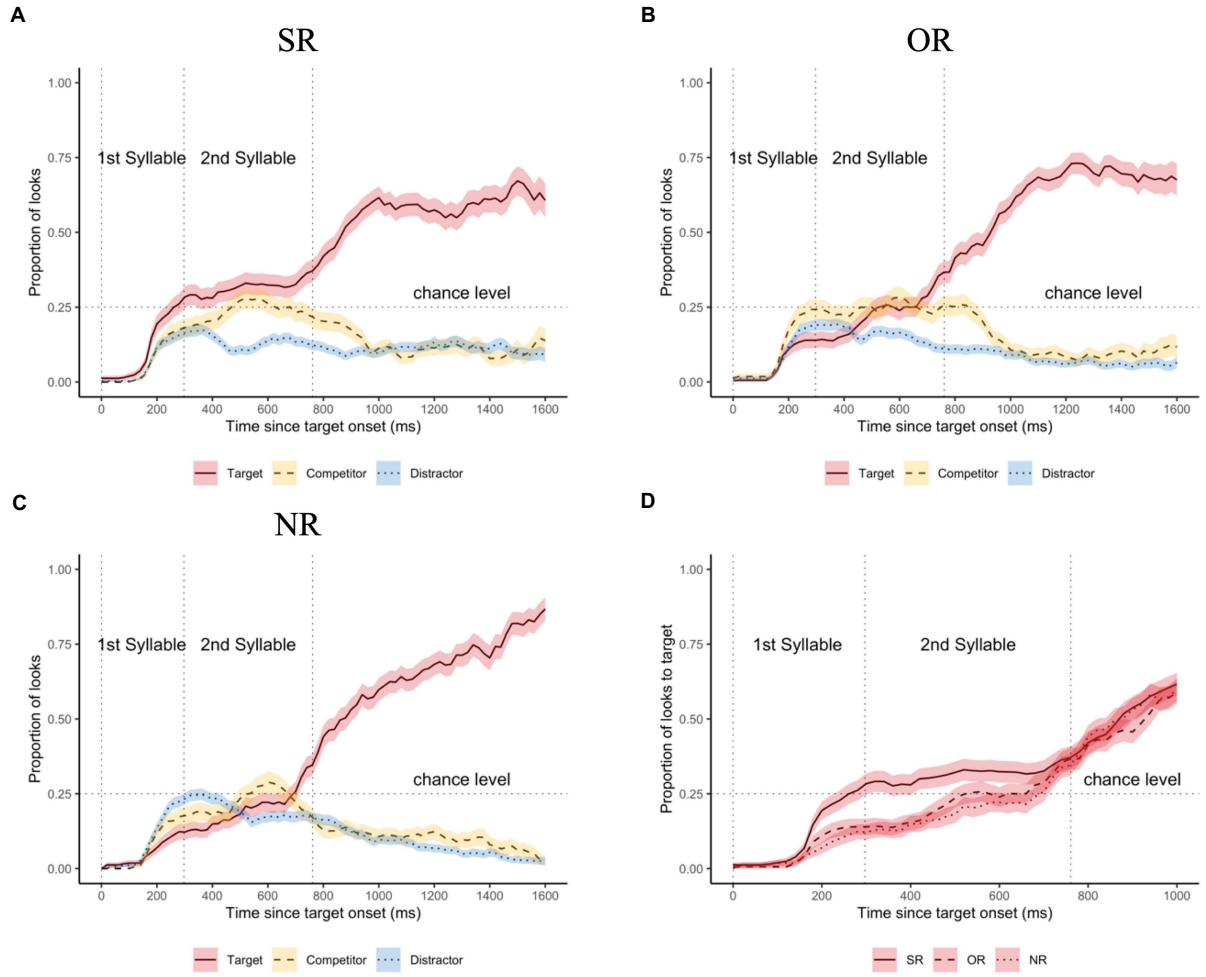
The mean proportion of looks in the three tonal coarticulation conditions. Panels **(A)**–**(C)** plot the proportions of looks to different visual objects until 1,600 ms since target onset in the SR condition **(A)**, the OR condition **(B)**, and the NR condition **(C)**, respectively. Solid lines indicate the mean proportions of looks to the targets (with red ribbons for the ±1 standard error); dashed lines for the mean proportions of looks to the competitors (with yellow ribbons for the ±1 standard error); dotted lines for the distractors (with blue ribbons for the ±1 standard error). Panel **(D)** plots proportion of looks to the target in the three conditions until 1,000 ms since target onset. Solid lines indicate the mean proportions of looks to target in the SR condition; dashed lines for the OR condition; dotted lines for the NR condition. Red ribbons for the ±1 standard error. Vertical lines indicate the mean syllable boundaries of the auditory stimuli, horizontal lines indicate the chance level.

Figure 3 illustrates the mean proportion of looks averaged across different participants and target items in three tonal coarticulation conditions, where Panels (A)-(C) plot the proportions of looks to different visual objects (i.e., target, competitor, distractor) in the SR condition, the OR condition, and the NR condition, respectively. To better illustrate the differences among the three conditions, Panel (D) plots the mean proportion of looks to target across different conditions.

As can be seen from Figures 3A–C, the mean proportions of looks remained at the bottom level in all three conditions until around 200 ms since target onset when the proportion curves started to increase. This period relatively corresponded to the time for planning and executing a saccadic eye movement (Matin et al., 1993), after which the proportion curves began to diverge. The mean proportion of looks to target exceeded the chance level shortly after 200 ms in the SR condition (see Figure 3A), relatively corresponding to the end of the first syllable on average. However, in the other two conditions (see Figures 3B,C), the proportions of looks to target increased in much slower rates, both of which remained below the chance level until after 600 ms, which is close to the end of the second syllable.

The mean proportions of looks to both competitor and distractor in all three conditions showed increasing patterns by the end of the first syllable. However, the difference between competitor and distractor is that, as soon as the proportion hit the chance level, curves for distractors soon fell to the bottom level (around 1%) across conditions, but those for competitors tended to stay around the chance level until the end of the entire stimulus. After around 1,000 ms, the proportion curves for competitors showed converging patterns with those for distractors, suggesting no competition effect after 1,000 ms.

As can be seen from Figure 3D, within the time window of 0–1,000 ms since target onset, the proportion of looks to target in three conditions show quite clear differences by the end of the entire disyllabic stimuli, especially between SR versus the other two conditions. The proportion of looks to target in the SR condition showed much faster rate of increase (i.e., during the second half of the first syllable) and higher overall proportion (i.e., above chance level typically during the second syllable) than those in the other two conditions. After the stimulus offset, the differences are less observable.

Two separate GCA models were thus run for the proportion of looks to target within the time windows of 0–300 ms (i.e., relatively corresponding to the first syllable) and 300–800 ms (i.e., relatively corresponding to the second syllable), respectively. The dependent variable was the proportion of looks to target aggregated over each 20 ms bin, and both full models contained the fourth-order TIME terms, TONAL CONDITION (three levels: SR, OR, NR), and their interactions in the fixed-factor structure. The random-factor structure included the by-SUBJECT random intercept on intercept and linear time terms, as well as the by-SUBJECT random slope on the effect of TONAL CONDITION on intercept and linear time terms. By-ITEM random effects were not included for the current dataset due to over-fitting. Parameter-specific estimation for both models were presented in Table 1.

**Table 1:** Pairwise parameter-specific estimation results for the time windows of 0–300 ms (A) and 300–800 ms (B).

		A. 0–300 ms				B. 300–800 ms			
		Estimate	SE	*t*	*p*	Estimate	SE	*t*	*p*
Intercept	OR vs. SR	−0.05	0.01	−4.85	<0.001	−0.08	0.03	−3.13	<0.01
	NR vs. SR	−0.06	0.01	−5.76	<0.001	−0.11	0.03	−4.02	<0.001
	NR vs. OR	−0.01	0.01	−0.91	0.37	−0.02	0.03	−0.89	0.37829
Linear	OR vs. SR	−0.18	0.04	−4.95	<0.001	0.23	0.08	2.80	<0.01
	NR vs. SR	−0.23	0.04	−6.40	<0.001	0.24	0.08	2.94	<0.01
	NR vs. OR	−0.05	0.04	−1.45	0.15	0.01	0.08	0.14	0.89
Quadratic	OR vs. SR	−0.06	0.03	−2.12	<0.05	0.01	0.04	0.19	0.85
	NR vs. SR	−0.06	0.03	−2.13	<0.05	0.08	0.04	1.84	0.07
	NR vs. OR	−0.01	0.03	−0.02	0.98	0.07	0.04	1.65	0.10
Cubic	OR vs. SR	0.02	0.03	0.59	0.55	0.01	0.04	0.04	0.97
	NR vs. SR	0.06	0.03	2.27	<0.05	0.03	0.04	0.74	0.46
	NR vs. OR	0.04	0.03	1.68	0.09	0.03	0.04	0.70	0.49
Quartic	OR vs. SR	0.01	0.03	0.26	0.79	0.01	0.04	0.22	0.83
	NR vs. SR	0.01	0.03	0.52	0.60	0.01	0.04	0.08	0.94
	NR vs. OR	0.01	0.03	0.26	0.80	−0.01	0.04	−0.14	0.89

As in Table 1, the proportion of looks to target showed significant differences between SR vs. the other two conditions in both time windows, while OR and NR did not significantly differ from each other in any term. In the 0–300 ms time window, the differences between SR vs. the other two conditions could be seen in almost all time terms except the quartic term. Among those, results of the intercept and linear terms (i.e., the negative intercept and linear estimates when conditions OR and NR were compared to SR in Table 1A) confirmed the observations that the mean proportion curve in the SR condition demonstrated a higher overall proportion and increased in a faster rate than those in OR and NR. However, in the 300–800 ms time window, only the intercept and linear terms between SR and the other two conditions were found to be significant. While the results confirmed the higher overall proportion level in the SR condition (i.e., the negative intercept estimates for both OR vs. SR and NR vs. SR in Table 1A), the rate of increase clearly slowed down compared to that in the other conditions (i.e., the positive linear estimates for both OR vs. SR and NR vs. SR in Table 1B).

## 4. Discussion

The present study set out to examine the participants’ eye movements within the VWP when they heard disyllabic stimuli with three tonal coarticulation conditions in Tianjin Mandarin, i.e., the SR condition (i.e., T2T1), the OR condition (i.e., T2T3), and the baseline NR condition (i.e., T2T4). The results yielded significant differences in the time course of online processing between the SR condition and the other two conditions.

Specifically, the participants initiated clearly more looks to the target for the first syllable (i.e., until around 300 ms) in the SR condition than in the other two conditions right after the saccadic latency of around 200 ms, and the rate of proportion increase for target during this period was also the fastest among all conditions. By the end of the first syllable, the proportion of looks to target in the SR condition has already exceeded the chance level (i.e., 25% for a four-option forced-choice task), suggesting a much higher level of activation for the first T2 in the SR condition.

During the second syllable (i.e., relatively corresponding to the 300–800 ms time window), the differences between SR and the other conditions remained, whereas the increase of the proportion curve in the SR condition slowed down and stayed in a relatively stable level that is slightly higher than 25%. The proportion of looks to target in the other two conditions tended to increase in a relatively linear way as that during the first syllable, so that the proportions did not exceed the chance level until the offset of the second syllable.

After the whole target stimulus has finished (i.e., after around 800 ms), participants seemed to be more confident about what has been just heard. The proportion of looks to target started to show an overwhelming advantage than looks to competitor or distractors in all three conditions. No competition effect between competitor vs. target was observed after around 1,000 ms, suggesting a complete activation and recognition of the target word.

The different time-course pattern in the SR condition thus indicated a perceptual benefit in anticipating what the upcoming tone should be due to the slope raising brought by the low-falling T1 in the second syllable. However, similar facilitatory effect was not observed for targets where the first T2 was raised in the overall-height by a following low-dipping T3, as no significant difference was found between the OR condition vs. the baseline condition of NR where no anticipatory raising effect was expected at all. The observed perceptual benefit due to anticipatory raising started immediately after participants made the first saccadic eye movements – i.e., around the mid-point of the first syllable in the auditory stimulus – and was maintained throughout the second syllable before the participants fully recognized the target word.

The results are therefore comparable with observations in segmental studies (e.g., Beddor et al., 2013) where listeners closely track the unfolding coarticulatory information (i.e., vowel nasalization), and actively utilize the nasalization information in anticipating the following nasal consonants before they actually hear them. In addition, this preliminary experiment also showed that, not all anticipatory tonal coarticulation effects triggered equal perceptual benefits. At least in the two types of anticipatory raising effects in Tianjin Mandarin, listeners seemed to be only sensitive to the slope raising in the perception of rising tone.

Such asymmetry in sensitivity of perceptual cues has also been observed in cross-linguistic studies of tone perception in connected speech, where listeners were also more sensitive to modifications of f0 directions than other cues such as the onset f0 height (e.g., Brunelle et al., 2016 with evidence from Hanoi Vietnamese) and the overall f0 mean (e.g., Qin and Zhang, 2022 with evidence from Macau Cantonese). As discussed in Qin and Zhang (2022), this could be attributed to the possibility that the f0 overall-height change is less salient than the slope change. As also seen from Figure 1, the time-normalized f0 mean of T2 in the OR condition does show relatively smaller deviation from that in the NR condition, as compared to that in the SR condition. However, it is also acknowledged that as the auditory target words were naturally produced, each target item shows much variabilities in the actual f0 realization and syllable duration within the same condition, leading to considerable noise in the eye movement data. Further research with better-controlled resynthesized stimuli are thus in need to investigate the role of different aspects of f0 variation. Moreover, due to the relatively small number of trials in the current experiment, the effect of visual stimulus location has introduced much noise to the eye-movement data especially for those of competitors and distractors, as in the current setup, only the locations of targets were counterbalanced, while those for competitors and distractors were not. More trials with better counterbalancing schemes were thus in great need in future studies.

Previous studies on the perception of lexical tones in connected speech have emphasized the importance of the contexts in the identification of tones (e.g., Xu, 1994) when they deviate from their canonical f0 realizations. On the one hand, our preliminary data have lent further support to this contextual effect as the listeners need to get access to the tonal contexts to achieve the complete activation and recognition of the target word. On the other hand, by extending this body of literature, our results showed, for the first time, that in the online processing of coarticulated tones, listeners do not just passively wait for the tonal contexts, but rather actively utilize the heard coarticulated cues and update the activation level of the lexical tones in real time before the tonal contexts are available. Moreover, while various studies have suggested the necessity of including canonical lexical tones in the existing models of spoken word recognition (e.g., Malins and Joanisse, 2010; Yang and Chen, 2022; Zou et al., 2022 for the TRACE model), the present study further implied that the contextual variation of lexical tones should also be adequately considered.

Taken together, with evidence from eye movements within the VWP, the present study showed that listeners are not completely relying on the tonal contexts in order to identify the lexical tones, especially those with f0 variation due to anticipatory tonal coarticulation in connected speech. Rather, listeners are incrementally processing the heard speech signals and actively utilizing the real-time anticipatory coarticulatory cues to recognize the coarticulated tones and anticipate the incoming tonal contexts.

## Data availability statement

The raw data supporting the conclusions of this article will be made available by the authors, without undue reservation.

## Ethics statement

Ethical review and approval was not required for the study on human participants in accordance with the local legislation and institutional requirements. The patients/participants provided their written informed consent to participate in this study.

## Author contributions

The author confirms being the sole contributor of this work and has approved it for publication.

## Funding

This research was funded by National Social Science Funds of China for Young Scholars (No. 19CYY021).

## Conflict of interest

The author declares that the research was conducted in the absence of any commercial or financial relationships that could be construed as a potential conflict of interest.

## Publisher’s note

All claims expressed in this article are solely those of the authors and do not necessarily represent those of their affiliated organizations, or those of the publisher, the editors and the reviewers. Any product that may be evaluated in this article, or claim that may be made by its manufacturer, is not guaranteed or endorsed by the publisher.
