# Supplementary material for: A preliminary study on the online processing of anticipatory tonal coarticulation – Evidence from eye movements
Source: Front Psychol. 2023 Apr 20;14:1137095. doi: 10.3389/fpsyg.2023.1137095 (PMC10157475; doi:10.3389/fpsyg.2023.1137095)
Supplement: Supplementary file 1 [file Data_Sheet_1.docx]

Supplementary Material

A Preliminary Study on the Online Processing of Anticipatory Tonal Coarticulation – Evidence from Eye Movements

# Supplementary Table 1. The stimuli used in the critical trials.

## The Slope Raising Condition

|  | Target | Competitor | Distractors | |
| --- | --- | --- | --- | --- |
| Character | 流星 | 流行 | 商店 | 报警 |
| Pinyin | liu^2^ xing^1^ | liu^2^ xing^2^ | shang^1^ dian^4^ | bao^4^ jing^3^ |
| Gloss | *meteor* | *popular* | *shop* | *to report to the police* |
| Character | 石阶 | 时节 | 餐厅 | 腰带 |
| Pinyin | shi^2^ jie^1^ | shi^2^ jie^2^ | can^1^ ting^1^ | yao^1^ dai^4^ |
| Gloss | *stone steps* | *season* | *restaurant* | *belt* |
| Character | 油烟 | 油盐 | 自动 | 监视 |
| Pinyin | you^2^ yan^1^ | you^2^ yan^2^ | zi^4^ dong^4^ | jian^1^ shi^4^ |
| Gloss | *cooking fume* | *oil and salt* | *automatic* | *to monitor* |
| Character | 船身 | 传神 | 医院 | 硬币 |
| Pinyin | chuan^2^ shen^1^ | chuan^2^ shen^2^ | yi^1^ yuan^4^ | ying^4^ bi^4^ |
| Gloss | *hull* | *lifelike* | *hospital* | *coin* |
| Character | 圆圈 | 源泉 | 木琴 | 跳伞 |
| Pinyin | yuan^2^ quan^1^ | yuan^2^ quan^2^ | mu^4^ qin^2^ | tiao^4^ san^3^ |
| Gloss | *circle* | *source* | *xylophone* | *parachute* |

## The Overall-height Raising Condition

|  | Target | Competitor | Distractors | |
| --- | --- | --- | --- | --- |
| Character | 杨柳 | 洋流 | 包裹 | 摊贩 |
| Pinyin | yang^2^ liu^3^ | yang^2^ liu^2^ | bao^1^ guo^3^ | tan^1^ fan^4^ |
| Gloss | *poplar and willow* | *ocean current* | *parcel* | *street vendor* |
| Character | 球网 | 球王 | 家电 | 车道 |
| Pinyin | qiu^2^ wang^3^ | qiu^2^ wang^2^ | jia^1^ dian^4^ | che^1^ dao^4^ |
| Gloss | *net (for a ball game)* | *ball king* | *domestic appliances* | *lane* |
| Character | 陈腐 | 沉浮 | 挂号 | 天鹅 |
| Pinyin | chen^2^ fu^3^ | chen^2^ fu^2^ | gua^4^ hao^4^ | tian^1^ e^2^ |
| Gloss | *hackneyed* | *rise and fall* | *to register* | *swan* |
| Character | 食指 | 时值 | 区号 | 松鼠 |
| Pinyin | shi^2^ zhi^3^ | shi^2^ zhi^2^ | qu^1^ hao^4^ | song^1^ shu^3^ |
| Gloss | *index finger* | *time value* | *area code* | *squirrel* |
| Character | 红眼 | 红颜 | 炼乳 | 钓鱼 |
| Pinyin | hong^2^ yan^3^ | hong^2^ yan^2^ | lian^4^ ru^3^ | diao^4^ yu^2^ |
| Gloss | *pinkeye* | *beautiful woman* | *condensed milk* | *fishing* |

## The No Raising Condition

|  | Target | Competitor | Distractors | |
| --- | --- | --- | --- | --- |
| Character | 词性 | 辞行 | 鸡腿 | 腊肠 |
| Pinyin | ci^2^ xing^4^ | ci^2^ xing^2^ | ji^1^ tui^3^ | la^4^ chang^2^ |
| Gloss | *part of speech* | *to bid farewell* | *drumstick* | *sausage* |
| Character | 咸菜 | 贤才 | 猪肉 | 墨鱼 |
| Pinyin | xian^2^ cai^4^ | xian^2^ cai^2^ | zhu^1^ rou^4^ | mo^4^ yu^2^ |
| Gloss | *salted vegetables* | *virtuous* | *pork* | *cuttlefish* |
| Character | 辞世 | 磁石 | 现金 | 热狗 |
| Pinyin | ci^2^ shi^4^ | ci^2^ shi^2^ | xian^4^ jin^1^ | re^4^ gou^3^ |
| Gloss | *to pass away* | *magnetite* | *cash* | *hot dog* |
| Character | 鱼翅 | 鱼池 | 豆浆 | 汽水 |
| Pinyin | yu^2^ chi^4^ | yu^2^ chi^2^ | dou^4^ jiang^1^ | qi^4^ shui^3^ |
| Gloss | *shark’s fin* | *fishpond* | *soya milk* | *soft drink* |
| Character | 茶室 | 查实 | 玉米 | 鹦鹉 |
| Pinyin | cha^2^ shi^4^ | cha^2^ shi^2^ | yu^4^ mi^3^ | ying^1^ wu^3^ |
| Gloss | *tea room* | *to check and verify* | *corn* | *parrot* |
